# Biosynthesized nanoparticles: a novel approach for cancer therapeutics

**DOI:** 10.3389/fmedt.2023.1236107

**Published:** 2023-07-13

**Authors:** Vivek K. Chaturvedi, Bhaskar Sharma, Abhay Dev Tripathi, Dawesh P. Yadav, Kshitij RB Singh, Jay Singh, Ravindra Pratap Singh

**Affiliations:** ^1^ Department of Gastroenterology, I.M.S., Banaras Hindu University, Varanasi, Uttar Pradesh, India; ^2^ Neurobiology Laboratory, Department of Anatomy, All India Institute of Medical Sciences, New Delhi, India; ^3^ School of Biochemical Engineering, Indian Institute Technology-BHU, Varanasi, Uttar Pradesh, India; ^4^ Department of Chemistry, Institute of Science, Banaras Hindu University, Varanasi, Uttar Pradesh, India; ^5^ Department of Biotechnology, Faculty of Science, Indira Gandhi National Tribal University, Amarkantak, Madhya Pradesh, India

**Keywords:** nanotechnology, cancer therapy, biogenic nanoparticles, biosynthesis, nanobiotechnlogy

## Abstract

Nanotechnology has become one of the most rapid, innovative, and adaptable sciences in modern science and cancer therapy. Traditional chemotherapy has limits owing to its non-specific nature and adverse side effects on healthy cells, and it remains a serious worldwide health issue. Because of their capacity to specifically target cancer cells and deliver therapeutic chemicals directly to them, nanoparticles have emerged as a viable strategy for cancer therapies. Nanomaterials disclose novel properties based on size, distribution, and shape. Biosynthesized or biogenic nanoparticles are a novel technique with anti-cancer capabilities, such as triggering apoptosis in cancer cells and slowing tumour growth. They may be configured to deliver medications or other therapies to specific cancer cells or tumour markers. Despite their potential, biosynthesized nanoparticles confront development obstacles such as a lack of standardisation in their synthesis and characterization, the possibility of toxicity, and their efficiency against various forms of cancer. The effectiveness and safety of biosynthesized nanoparticles must be further investigated, as well as the types of cancer they are most successful against. This review discusses the promise of biosynthesized nanoparticles as a novel approach for cancer therapeutics, as well as their mode of action and present barriers to their development.

## 1. Introduction

Tumours are benign or malignant masses of tissue that may present in the form of sarcomas, carcinomas and lymphomas depending on their infected origin. The most often utilized cancer therapeutic options typically combine surgical, chemotherapy and radiotherapy (1). Surgical excision therapy is by using a sharp cutting instrument (scalpel) to extract the tissue mass or growth. Combining it with chemotherapy and radiation therapy has boosted the surgery’s efficacy in recent years (2). Radiation destroys cancer cells by breaking the DNA sequences and generating hydroxyl free radicals. Extensive DNA damage triggers apoptosis and cell death (3). Chemotherapy treatment kills or stops the growth of malignant fast-growing cells by directly blocking DNA replication and cell division and activates programmed cell death in tumour cells (4). Hormonal therapy is called endocrine therapy, anti-hormonal therapy in which, hormones are blocked or removed to delay or stop cancer cell development (5). A stem cell transplant may be used to treat various malignancies to more effectively destroy cancer cells (6). However, despite these advances, cancer remains a major health concern, with millions of people worldwide being diagnosed with the disease each year.

The application of biosynthesized or biogenic nanotechnology in cancer therapy is one potential field of study that has attracted growing attention in recent years. Biogenic nanoparticles are tiny particles produced by living organisms, such as viruses or bacteria. These particles can be engineered to target cancer cells specifically, imaging, sensing and delivering drugs or other therapeutic agents straight to the tumour site. The capacity of biogenic nanotechnology to precisely target cancer cells is one of its main advantages. This means that less of the drug is required, reducing the risk of side effects and toxicity. The capability of biogenic nanoparticles to target cancer cells can also be improved by giving them the capability to react to particular stimuli, like changes in temperature or pH levels. One approach to treating cancer with biogenic nanotechnology is the use of nanoparticles functionalized with molecules that bind specifically to cancer cells, enabling targeted drug delivery. These innovations have a great deal of potential to improve patient quality of life, lessen the adverse effects of traditional cancer therapies, and improve cancer outcomes.

## 2. Types and properties of biogenic nanomaterials

Biogenic nanoparticles, which are produced by living things like plants, bacteria, fungi, and algae, offer unique properties including low toxicity, biocompatibility, and environmental friendliness that make them a desirable option for medical applications. Various types of biogenic nanoparticles with different properties can be described. Plants can be used to synthesise nanoparticles, such as green tea, garlic, and ginger. Silver nanoparticles made from green tea extract have powerful antioxidant, antibacterial, and anticancer properties, while ginger extract has been used to create gold nanoparticles with anticancer efficacy against breast cancer cells (7). Bacteria are another source of biogenic nanoparticles, which are distinguished by their small size and structure. Nanoparticles have been produced using several microbes, including Escherichia coli, Pseudomonas aeruginosa, and Bacillus subtilis. In addition to having potent antibacterial and antifungal capabilities, these nanoparticles also exhibit anticancer effects on ovarian cancer cells (8). Secondary metabolites and enzymes useful in the production of nanoparticles can be found in large quantities in fungi. Fungal nanoparticles have unique features like biocompatibility and low toxicity, making them a promising candidate for biomedical applications. Some of the most often employed fungi are Aspergillus, Penicillium, and Fusarium. Silver nanoparticles produced using Fusarium oxysporum have a significant anticancer effect against breast cancer cells (9). Algae are photosynthetic organisms that can be used to synthesise nanoparticles, which have great stability, biocompatibility, and minimal toxicity. Spirulina, Chlorella, and Dunaliella are three of the most often employed algae for nanoparticle manufacturing, with gold nanoparticles producing significant anticancer effects against liver cancer cells (10). Due to their unique physicochemical features, biocompatibility and simplicity of modification, biogenic nanoparticles can be programmed to target cancer cells. Several other nanoparticles that potentially show promising effects in cancer therapy are discussed in this review.

One such nanoparticle is gold, having anticancer properties by binding to cancer cells and disrupting cellular signalling pathways, leading to cell death (11). Through oxidative stress, DNA damage, and rupture of cell membranes, silver nanoparticles exert lethal effects on cancer cells (12). Metal oxide nanoparticles, like gold and silver, between 1 and 10 nm in diameter can function as catalysts and are among the most alluring categories of cancer treatment agents. These nanoparticles scatter samples of visible light *in vitro*, acting as contrast media, and can be employed for biopsies as well as the diagnosis of pancreatic and cervical malignancies. They are also used as a diagnostic tool for many tumours and have numerous uses in a variety of industries, including imaging, diagnoses and biosensing. Iron oxide nanoparticles are utilised in magnetic resonance imaging (MRI) and targeted medication delivery for cancer treatment (13).

Through oxidative stress and reactive oxygen species (ROS), zinc oxide nanoparticles trigger death in cancer cells (14). Copper nanoparticles have anticancer properties, while platinum nanoparticles have been used as a chemotherapeutic agent with the ability to inhibit DNA replication and induce apoptosis (15). Anti-cancer properties of Palladium nanoparticles are mainly because of their ability to induce DNA damage and inhibit cell proliferation (16). Selenium nanoparticles can prevent the development and progression of cancer with their antioxidant and anti-inflammatory properties (17). Titanium dioxide nanoparticles can encourage apoptosis in cancer cells through ROS generation (18). Calcium carbonate nanoparticles act to trigger apoptosis in cancer cells and to prevent angiogenesis (19). Fullerenes were first used in 1985, and the first investigational evidence for it was uncovered, opening up the possibility for research into its potential applications in healthcare, photovoltaics, and other fields (20). Because of their potential to encapsulate and transport medications directly to cancer cells, chitosan, protein, and lipid nanoparticles can penetrate cell membranes and provide specific drug administration for cancer treatment (21, 22).

Magnetic nanoparticles have also been used to induce heat and destroy cancer cells (23). Carbon nanotubes (CNTs) are used in photothermal therapy for cancer therapy (24). CNTs are cylindrical materials with a nanometre-sized thickness and axial uniformity that are useful in the detection and therapy of cancer. They may be categorised as single-walled and multi-walled CNTs based on their shape and size. The architecture, surface area, good mechanical, and metallic activity, thermal conductivity, electrical conductivity, and ultra-lightweights of CNTs are all related to their physical and chemical characteristics, making them a good contender for many biological purposes. Due to its capacity to absorb and transform light energy into heat, graphene nanoparticles can be used in the photothermal treatment and targeted medication delivery (25). Quantum dots are used for imaging in cancer therapy due to their ability to emit fluorescent light (26). Quantum dots (QDs) were created in 1980, which are tiny nanoparticles or semiconductor nanocrystals with a dimension between 2 and 10 nanometres. QDs are a potential tool for determining tumour biomarkers, such as membrane proteins or other elements of diverse tumour samples.

Cerium oxide nanoparticles have antioxidant properties, which may help prevent the development and progression of cancer (27). Lanthanide nanoparticles show unique optical properties and are used for imaging purposes (28). Calcium phosphate nanoparticles have been used in gene delivery for cancer therapy to protect the degradation of genetic material and deliver it directly to cancer cells (29). Hydroxyapatite nanoparticles enhance the targeted drug delivery as they can bind to cancer cells (30). Polymeric nanoparticles are biocompatible and show the ability to encapsulate and deliver drugs directly to cancer cells (22). One such polymer is Dendrimers, which are highly branched, synthetic polymers which can encapsulate drugs and deliver them directly to cancer cells, or be functionalized with targeting moieties such as antibodies, peptides, or aptamers to improve drug delivery efficiency (31). Solid lipid nanoparticles (SLNs) are lipid-based nanoparticles used in cancer therapy to encapsulate hydrophobic drugs and improve their solubility, stability, and bioavailability. Additionally, because of their amplified drug-loading capacity and ability to release medications over time, they have greater pharmacological effectiveness and less toxicity (32).

Nanoemulsions are oil-in-water emulsions with droplet sizes in the nanometre range used in cancer therapy to improve drug solubility, bioavailability, and stability. To target cancer cells specifically and improve the effectiveness of medication delivery, they can also be functionalized with targeting moieties (33). Nanogels are cross-linked polymeric nanoparticles used in cancer therapy to encapsulate drugs and release them in a precise and persistent manner, resulting in improved drug efficacy and reduced toxicity. To target cancer cells specifically and increase the effectiveness of medication delivery, nano gels can also be functionalized with targeting moieties (34). Mesoporous Silica nanoparticles (MSNs) are porous silica nanoparticles with a large surface area and the capacity to encapsulate medications that are utilised in cancer treatment (35). Self-assembling nanoparticles are nanoparticles that spontaneously assemble into larger structures in response to certain stimuli. They have been used in cancer therapy due to their ability to improve drug solubility, bioavailability, and stability. They can also be functionalized to improve drug delivery efficiency (36). DNA and RNA nanoparticles are used in cancer therapy to deliver therapeutic genes directly to cancer cells, which improves the therapeutic efficacy and reduces toxicity (37, 38). Due to their distinctive physical and chemical characteristics, biologic nanoparticles offer a great deal of potential for cancer therapy; nevertheless, further study is required to fully comprehend their modes of action and maximise their therapeutic potential.

## 3. Drawbacks of conventional nanomaterial synthesis

### 3.1. Conventional techniques for creating nanoparticles

Nanoparticles can be created via physical, chemical, photochemical, and biological processes. Methods of dispersion and condensing can be employed for colloidal nanoparticles. Condensation methods are based on a chemical reaction, whereas dispersion methods depend on the breakdown of the material’s crystal lattice.

#### 3.1.1. Dispersion method

Laser Ablation: The technique is based on the pulsed laser irradiation of a silver sheet submerged in a surfactant or water solution.

Vacuum spitting: This method is based on the use of an electric field to apply a potential difference between the two electrodes in a vacuum chamber. The chamber receives an inert gas, which is then ionised. An argon plasma bombardment of a metal target (cathode) occurs. Atomic clusters are subsequently punched out of the target region and placed on the surface or into a liquid solution. The ease and purity of the procedure make this approach one of the most favoured ones.

#### 3.1.2. Condensation methods

There are several aqueous (solution or wet) techniques available today for the synthesis of gold nanoparticles, which vary depending on the experimental circumstances and enable the production of nanoparticles with the appropriate form and dispersion. The production of smaller nanoparticles is often ensured by a large reduction force, which also guarantees a rapid response rate. Large nanoparticles arise as a result of weak reducing agents’ slow reaction rate. Additionally, the form of nanoparticles is significantly influenced by reducing agents.

1. Solution Diminishment: By using reducing agents like sodium citrate, which was originally employed for gold nanoparticles, this technique allows for the production of spherical nanoparticles with a size range of 5–40 nm. Later, silver nanoparticles were prepared using this process, however the spread of the silver nanoparticles produced by this method was wider, ranging from 60 to 200 nm. As a reducing agent and stabiliser, the citrate anion complicates the choice of the ideal citrate ratio and has a significant impact on the nucleation and particle development processes, which is a drawback of this approach. Additionally, additional substances like hydrogen peroxide, sodium borohydride (NaBH_4_), or ascorbic acid have been effectively utilised as reducers in the creation of nanoparticles.
2. The Brust-Schiffrin Approach: In a two-phase aquatic-organic environment, it represents the production of hydrophobic gold clusters, 1–3 nm in size, stabilised by an alkanethiol monolayer. The spatial separation of nanoparticles into two immiscible phases is the goal of the synthesis. Diffusion, a process phase that has a rate restriction, is prevented by the organic layer. The interface (aqueous-organic media) is where the reaction rate is constrained. The creation of an alkanethiol monolayer, which is situated in a non-polar environment, is what causes separation and hydrophobization. Tetra-n-alkylammonium was utilised as the interfacial carrier, while toluene was used as the non-polar medium by Brust and Schiffrin. The Brust-Schiffrin technique may also be used to synthesise silver nanoparticles.
3. Micelle Reverse Synthesis: This technique enables the creation of nanoparticles in a small area while also allowing for growth control. An emulsifier and a surfactant were utilised to create the micro emulsion that was used in this procedure, which is a thermodynamically stable dispersion of two immiscible liquids. Organic compounds with varying polarity make up surfactants. The molecule is made up of two parts: a polar component and a non-polar, hydrophobic chain of hydrocarbons. Nanoparticles develop in the polar core of the micelle as opposed to reduction in molecular solution. To create metallic nanoparticles, they employ the water-in-oil technique. In this instance, a liquid organic solvent is used to distribute the water. Micelles may be built as a unipolar surface embellished with gold nanoparticles in addition to being utilised to create nanoparticles.
4. Ultraviolet-Based Process: By exposing aqueous solutions containing AgClO4 or NaAuCl4, acetone, 2-propanol, and different polymeric stabilisers to UV light, silver nanoparticles can be created. Ketyl radicals are produced when UV light excites acetone. For the creation of silver nanoparticles utilising UV radiation, many methods have been used. Chloramine T was used as an organic modification to create ultrasmall AgNPs with various optical characteristics. For changes in mobility and dissolution of citrate-coated silver nanoparticles, ultraviolet (UV) irradiation with UVA (320–400 nm) or UVB (280–320 nm) beam was utilised. For the quantitative measurement of uric acid, cholloidoidal silver nanoparticles were also made using the UV-light-induced citrate reduction approach. Pullulan-mediated silver nanoparticle green synthesis has also been accomplished using ultraviolet light.

The typical drawbacks of conventional techniques are readily apparent in the instance of ball milling, which is neither cost-effective nor business-friendly because it requires a lot of time and energy to generate nanomaterials (39–41). Continuous laser ablation, like the other top-down technique, needs a lot of energy input to create a powerful enough laser (42). While hydrothermal and microwave processes among the bottom-up options demand a costly autoclave and intricate equipment, they are commercially unviable despite the fact that vapour deposition also consumes a lot of energy (43). The employment of additional treatment techniques is necessary due to the usage of various compounds in chemical reduction, including sodium borohydride, hydrazine, and N, N-dimethylformamide, all of which are very harmful to both living creatures and the environment (44, 45). With all of the aforementioned problems, numerous researchers have looked into environmentally friendly synthesis techniques to find more biologically acceptable substitutes for creating nanomaterials.

## 4. Synthesis of biogenic nanoparticles

Green synthesis techniques, such as those based on bacteria, fungi, algae, and plants, use natural or biological components to create nanomaterials that are safe, non-harmful, and environmentally friendly. Benefits include lowering energy requirements, eliminating toxic chemicals and being affordable. Bacteria are plentiful, easy to cultivate, inexpensive, stable, and simple to regulate. Bacteria can convert toxic metal ions into non-toxic metal oxide nanomaterials, justifying using them in nanomaterial production.

**Figure 1:**
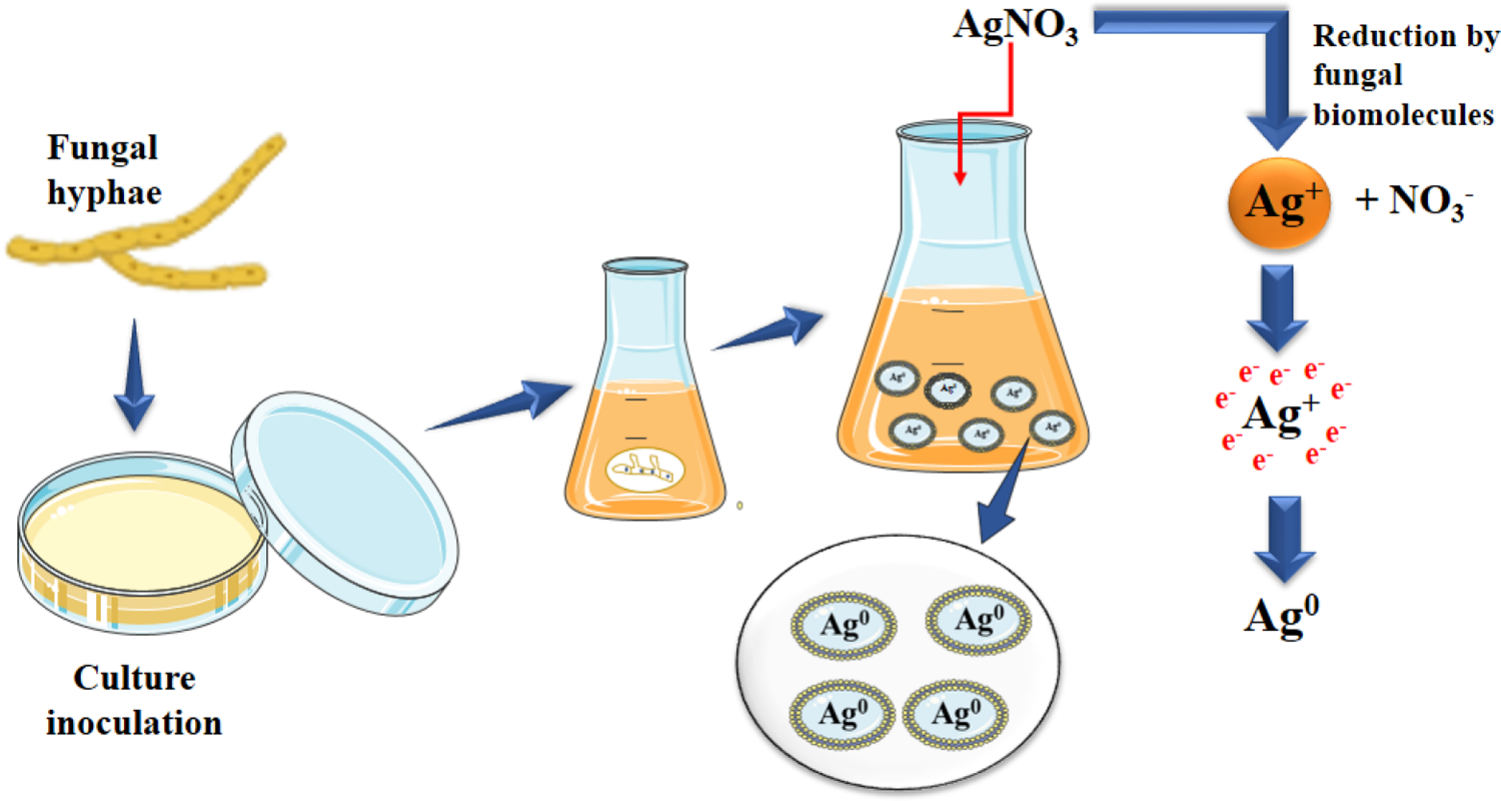
Fungal hyphae mediated green synthesis of Ag nanoparticle: fungal biomolecules act as a reducing agent on AgNO_3_ which leads to the synthesis of Ag NP.

Given their capacity for internalisation, bioaccumulation, and tolerance to heavy metals, fungi make great candidates for use as reducing and stabilising agents in the production of metal nanoparticles (Figure 1). They can also be generated in large numbers and generate more proteins and enzymes than bacteria, allowing them to provide better synthesis productivity. Algal-mediated synthesis of nanomaterials uses minerals, lipids, proteins, carbohydrates, and bioactive substances as reducing agents. Algae are ideal candidates due to their capacity for hyperaccumulating heavy metals, and the extracellular or intracellular processes used in algae-mediated nanomaterial creation can provide appropriate regulation over production parameters. The application of microorganism-mediated synthesis processes is currently hampered by the pathogenic traits of organisms, underlying safety concerns, and a lack of understanding of the synthesis mechanisms.

Plants are well-suited for nano synthesis, as they do not have harmful effects and their nanomaterials are more homogeneous than those produced by other methods. Plant extracts contain stabilising chemicals that can cling to nanoparticle surfaces, improving surface reaction kinetics and particle stability. Plant-mediated metal nanoparticle production is restricted to the reduction of a precursor salt in the presence of a metal ion precursor. Amino acids, proteins, vitamins, terpenes, flavones, amides, saponins, phenolics, and polysaccharides are examples of phytochemicals that act as reducing and stabilising agents in nanomaterials (46, 47).

Biogenic nanoparticle synthesis for cancer therapy involves the use of nanoparticles with specific properties that allow them to target and destroy cancer cells. The general procedure for creating biogenic nanoparticles for cancer treatment is as follows:

1. Selection of biological agent: The first step in the biogenic nanoparticle synthesis process is the selection of a suitable biological agent. For example, plant extracts, fungi, bacteria, and other natural sources may be used.
2. Preparation of biological extract: The selected biological agent is processed to obtain an extract that contains the active compounds necessary for nanoparticle synthesis. This may involve grinding or blending the biological material and then extracting it using solvents or water.
3. Reduction of metal ions: A mixture of metal ions, such as gold, silver, or platinum, is added to the extract. The extract’s active ingredients work as reducing agents to turn the metal ions into nanoparticles.
4. Characterization of nanoparticles: The size, shape, and stability of the nanoparticles are then determined using methods like UV-Vis spectroscopy, dynamic light scattering, and transmission electron microscopy.
5. Functionalization of nanoparticles: The nanoparticles may be functionalized with targeting molecules such as antibodies or peptides to improve their specificity for cancer cells.
6. In vitro and *in vivo* tests: The created nanoparticles are subsequently put to the test *in vitro* and *in vivo* to determine how well they target and eliminate cancer cells (48).

### 4.1. Silver and gold nanoparticle biosynthesis

For the production of non-toxic biocompatible nanoparticles, environmentally friendly nanoparticle development is required. Biosynthesis of nanoparticle requires some biological agent, which may be derived from plant, bacteria, fungi, etc. Using the bacterial strain Pseudomonas stutzeri AG259, it has been discovered that intracellular synthesis results in the production of polyhedral silver crystals with an average size of 100–200 nm during cell growth in the presence of Ag + ions, in the periplasmic region of the cells. Although the exact method of creation has not been determined, it has been hypothesised that proteins with an affinity for silver, some of whose components can serve as nucleation centres, play a part.

During the development of Verticillium AAT-TS-4, silver nanoparticles with an average size of 25 12 nm were produced as a result of intracellular production. Although the silver nanoparticles were immobilised in the cell walls, a theory was put up on the extracellular process of silver nanoparticle synthesis. By reducing Ag + ions with cell wall protein, metal clusters were created. Aqueous silver nitrate solutions may be intracellularly reduced by Fusarium oxysporum fungus to form metal nanoparticles with a diameter of 20–50 nm. At 415–420 nm, nanoparticles showed absorbance, and their aqueous dispersions showed stability over many days.

## 5. Biogenic nanoparticles for cancer treatment

Biogenic nanoparticles have been proven to have anticancer potential, but the exact mechanism is yet unknown. Depending on the phytochemical composition and interaction with the cancer cell, different nanoparticles exhibit different approaches. The nanoparticles are improved anticancer medicines due to the coating or binding of phytochemicals as functional groups, which also surges the rate of apoptosis (programmed cell death) in cancer cells (49). By adhering to surfaces and cellular absorption, the biogenic nanoparticles have anticancer characteristics in two different ways (50). Some nanoparticles, due to their low stability, break down into their ionic forms in the body fluid after delivery (51), whereas stable nanoparticles reach the cancer cells they are intended to treat (52). The ions dissociated in body fluid also have anticancer properties, but they are less specific to cancer cells. In physiological fluids, unstable nanoparticles are nano-encapsulated or nano-formulated to improve stability (53). Nanoparticles are the key factor in the suppression of malignant cells by creating ROS on the surface or entering cells to disrupt cell membranes (54). Biogenic nanoparticles can cause membrane protein degradation, transmembrane electron transport disruption, and cancer cell apoptosis (55).

The key advantage of utilising phytochemicals to create nanoparticles is that they are easier to internalise into cancer cells (56). When nanoparticles are internalised by cancer cells, there may be an increase in oxidative stress and osmotic pressure, which will lead to apoptosis (57). However, by employing nanoparticles, synergistic approaches combine two or more of the aforementioned processes to inhibit the growth of cancer cells (58). Additionally, nanoparticles may dissolve, allowing ions to enter cells through ion channels (59), or they may be drawn to cancer cells by electrostatic attraction (60). Due to their increased surface area to volume ratio and capacity to identify a variety of cancer biomarkers, nanoparticles are crucial for the diagnosis of cancer (61). They are incredibly responsive to even the smallest surface changes when used with biosensors, immediately sending signals to the sensors. For the rapid and safe detection of biomarkers in biofluids including sweat, blood, and urine, it is imperative to use biological nanoparticles coated with particular receptors. Further research is needed to specify and standardise nanoparticle anticancer mechanisms to increase their anticancer capacity (62).

**Figure 2:**
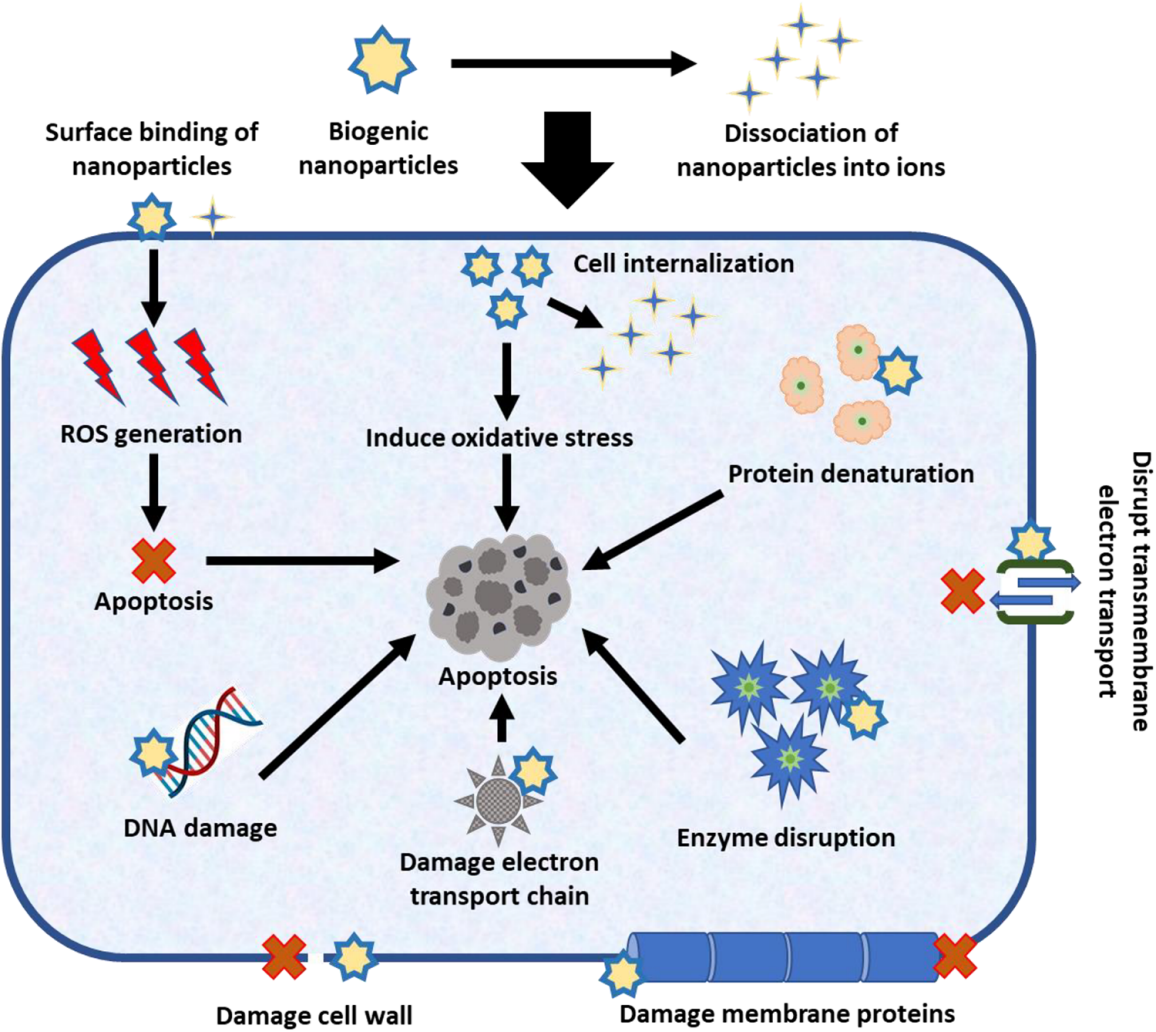
Anticancer mechanism of biogenic nanoparticles.

Two putative anticancer processes shared by the majority of biogenic nanoparticles are shown in the Figure 2. The first approach to treating cancer entails directly suppressing cancer cells with nanoparticles, either by rupturing their cell walls and membrane proteins, obstructing transmembrane electron transport, or by releasing reactive oxygen species (ROS) that promote apoptosis. Additionally, smaller nanoparticles may enter the cancer cell, produce ROS, lead to oxidative stress, affect DNA, the electron transport chain, enzymes, and proteins, and finally cause the cancer cell to undergo apoptosis. The second technique involves isolating nanoparticles into ions and adding anticancer activity to the isolated nanoparticle ions.

Due to the use of phytochemicals in their synthesis and the inclusion of plant extracts as functional active groups, nanoparticles have exceptional fluorescence properties that have been enhanced (63–65). They bind to cancer cells, internalise through the tagged receptors, and exhibit fluorescent features when they are labelled with particular cancer biomarker receptors. It is possible to encapsulate conventional fluorescent dyes with biogenic nanoparticles for targeted delivery into cancer cells with no negative effects on healthy cells. Multimodal cancer therapy methods frequently employ magnetic nanoparticles, which can be remotely driven inside the patient’s body using magnetic force to reach the desired location. Nanoparticles’ theranostic (detection and inhibition) mechanisms have recently drawn a lot of interest and are viewed as the cancer treatment of the future.

Biogenic magnetic nanocomposites will be coated with biomolecular receptors to bind with particular cancer biomarkers and either provide signals for biosensors or cancer cell bioimaging to find and detect the type of cancer and its location. Based on therapeutic efficacy and the development of malignant cells, drug release can be planned and carried out using an external magnetic field, making them a versatile cancer treatment that can be employed as a theranostic agent to treat cancer. Recent research by Kesavan et al. (2018) demonstrates the potential of biogenic nanocomposites in the treatment of cancer. Through the use of plant-derived saponins as bio-surfactants for cancer-targeted treatment, they created unique iron oxide-drug nanocage theranostic nanocomposites (66).

## 6. Conclusion

In conclusion, the future of cancer therapy with biogenic nanoparticles is bright. Biogenic nanoparticles have various advantages over chemically manufactured nanoparticles, including being more environmentally friendly, less expensive, and biocompatible. Biogenic nanoparticles can also be easily functionalized with targeting ligands or medicines, enabling precise targeting and delivery to cancer cells. Biogenic nanoparticles have shown promise for cancer therapy through a variety of methods, including apoptosis induction, angiogenesis suppression, and tumour microenvironment manipulation. Silver nanoparticles, gold nanoparticles, iron oxide nanoparticles, and zinc oxide nanoparticles have all demonstrated potential for cancer therapy. We should expect to see more tailored and targeted cancer therapeutics using biogenic nanoparticles in the future. Based on the unique cancer kind and genetic profile of each patient, biologic nanoparticles can be modified to provide more effective and individualised treatments. Biogenic nanoparticles can also be used in immunotherapy and combination therapy, which could increase effectiveness and minimise side effects. To increase medicine efficacy, lessen adverse effects, and improve patient outcomes, biogenic nanoparticles can be designed to transport and distribute therapeutic molecules to certain cells or tissues. Magnetic resonance imaging (MRI), computed tomography (CT), and optical imaging all benefit from the use of biological nanoparticles as contrast agents. In order to improve contrast and enable more precise visualisation of tissues and organs for the early identification of illnesses and infections, imaging molecules can be attached to nanoparticles. To aid in tissue regeneration and repair, they can be utilised to distribute growth factors, cytokines, or genetic material. For tissue engineering, biogenic nanoparticles can be used as scaffolds or templates, offering structural support and encouraging cell development and differentiation. A potential replacement for traditional antibiotics, biologic nanoparticles provide a solution to the worldwide problem of antimicrobial resistance. Biogenic nanoparticles can increase antigen presentation, boost the immune response, and encourage focused immune activation against certain illnesses. Overall, biogenic nanoparticle cancer therapy has a promising future, and additional studies in this area may provide significant advances in the diagnosis, treatment, and management of cancer.

## 7. Future perspective

The issue with the majority of the current chemical and physical techniques of producing nanomaterials is that they are very expensive and also require the use of toxic, dangerous substances, which might pose threats to the environment and human health. Consequently, there is a need for an ecologically friendly and practical method to create these nanoparticles. This opened the door for the safe manufacture of these nanoparticles using biomimetic methods. As a theragnostic agent, nanoparticles are often employed in the medical profession. Additionally, biogenic nanoparticles are used as biomarkers, bio delivery vehicles for medical treatments, anti-aging components, and biosensors. Nanoparticles made by biological synthesis have shown to be safe drug delivery systems for the treatment of cancer cells. Researchers’ ability to create safer nanomaterials and advance knowledge of the health and safety implications of nanoparticles will be aided by the biosynthesis of nanoparticles. Because the biomaterial-based approaches do not require the use of hazardous chemicals, useful products may be manufactured readily even at acceptable size. In order to be more environmentally friendly, single-pot processes without the inclusion of surfactants, capping agents, or templates have often been utilised in the synthesis of nanoparticles. As biosynthesised nanoparticles have significant applications, we think there are tremendous potential for establishing industrial-scale manufacturing.
